# Root Canal Cleaning Efficacy of Rotary and Hand Files Instrumentation in Primary Molars

**Published:** 2009-04-17

**Authors:** Kiumars Nazari Moghaddam, Majid Mehran, Hamideh Farajian Zadeh

**Affiliations:** 1 *Department of Endodontics, Dental School, Shahed University of Medical Sciences, Tehran, Iran*; 2 *Department of Pediatrics, Dental School, Shahed University of Medical Sciences, Tehran, Iran*; 3 *Dentist, Private Practice, Tehran, Iran*

**Keywords:** Cleaning Efficacy, Deciduous, Flexmaster, Instrumentation, Molar, Ni-Ti, Pulpectomy

## Abstract

**INTRODUCTION:** Pulpectomy of primary teeth is commonly carried out with hand files and broaches; a tricky and time consuming procedure. The purpose of this *in vitro* study was to compare the cleaning efficacy and time taken for instrumentation of deciduous molars using hand K-files and Flex Master rotary system.

**MATERIALS AND METHODS:** In this study, 68 canals of 23 extracted primary molars with at least two third intact roots and 7-12 mm length were selected. After preparing an access cavity, K-file size #15 was introduced into the root canal and India ink was injected with an insulin syringe. Sixty samples were randomly divided in to experimental groups in group I (n=30), root canals were prepared with hand K-files; in group II (n=30), rotary Flex Master files were used for instrumentation, and in group III 8 remained samples were considered as negative controls. After clearing and root sectioning, the removal of India ink from cervical, middle, and apical thirds was scored. Data was analyzed using student's T-test and Mann-Whitney *U* test.

**RESULTS:** There was no significant difference between experimental groups cleaning efficacy at the cervical, middle and apical root canal thirds. Only the coronal third scored higher in the hand instrumented group (P<0.001). Instrumentation with Flex Master rotary files was significantly less time consuming (P<0.001).

**CONCLUSION:** Although there was no difference in cleanliness efficacy at the apical and middle thirds, the coronal third was more effectively cleaned with hand files. Predictably, time efficiency was a significant advantage with rotary technique.

## INTRODUCTION

One of the most important concerns in pediatric dentistry is loss of necrotic primary molars leading to space loss. Although the morphology of root canals in primary teeth renders endodontic treatment difficult (1); pulpectomy of primary teeth with severe pulpal involvement should be considered as a treatment of choice. Clinical success occurs when the tooth is painless, firm, non-mobile, and without any signs of inflammation or infection. Radiographically, lesions should be resolved within six months, and no pathologic root resorption should be observed (2).

Bacteria and their by-products play an essential role in the initiation and perpetuation of pulpal and periapical disease (3). The primary objectives of cleaning and shaping the root canal system are removing soft and hard tissue containing bacteria, providing a path for irrigants to the apical third, supplying space for medicaments and subsequent obturation, retaining the integrity of radicular structure (2). Thus, success of pulpectomy depends on elimination of irritants by means of cleaning and shaping the root canal (4).

Root canal preparation is performed with files, reamers, burs, sonic instruments or mechanical apparatus, and with nickel-titanium (Ni-Ti) rotary file systems. Since most hand preparation techniques are time consuming and may lead to iatrogenic errors (*i.e.* ledging, zipping, canal transportation and apical blockage) (5), much attention has been directed toward root canal preparation techniques with Ni-Ti rotary instruments. Numerous studies have reported that they could efficiently create smooth, predetermined funnel-form shapes, with minimal risk of ledging and transportation (3,6-8).

Rotary instrumentation in curved molar root canals of permanent teeth has been shown to be time efficient, with increased patient comfort lower risk of flare-up (9,10).

Ni-Ti files do not need precurvature due to their elastic memory; they are motor-activated and can prepare the root canal with high speed. The probability of root canal deformation is reduced due to its elastic memory and radial land that keeps the file in the center of the root canal via wall support and inactive tips (11,12).

Although shaping procedures can be more easily and predictably completed, effective cleansing of the entire root canal system using Ni-Ti rotary instruments has not been demonstrated (3). The basic dilemma is that all rotary instruments are centered in root canals during rotation and leave unclean areas and potentially infected tissue in fins and isthmuses (13).

Despite advantages of rotary instrumentation and studies performed on primary molars, there are no clear guidelines or instructions for the suitable preparation technique of these teeth. Some authors believe in similar principles of rotary instrumentation for permanent and deciduous teeth (14,15). Barr *et al.* used Ni-Ti Profile® 0.04 taper rotary instruments for primary root canal preparation and concluded that the use of Ni-Ti rotary files for root canal preparation in primary teeth was cost-effective and rapid resulting in consistently uniform and predictable obturation (14). However, other studies found clinical success in primary molars with a modified protocol using Pro Taper files (16,17). Kuo *et al.* concluded ProTaper Ni-Ti rotary files can be safely and efficiently applied for root canal preparation in primary molars as well as using NaOCl for root canal irrigation (16).

Flex Master files have round, passive tips, a modified cross-section, convex triangular shape with sharp cutting edges and no radial lands; which resembles K-file configuration (enhancing dentine cutting effectiveness (2)).

Available Ni-Ti rotary files are designed mostly for conical root canal shapes.

However, most of the primary molar root canals are ribbon-shaped. Little is known about the impact of these design modifications on cleaning efficacy and the time involved for deciduous pulpectomy with rotary files. The aim of this experiment therefore, was to compare the cleaning efficacy and time efficiency of Flex Master rotary files and K-hand files.

## MATERIALS AND METHODS

Twenty three (10 maxillary and 13 mandibular) extracted primary molars with at least two-thirds of intact root, and 7-12 mm length were cleaned in water and stored in 0.5% sodium hypochlorite for 1 week. Radiographs were taken and 68 mesial and distal roots were selected. Coronal access was made with spherical diamond burs (Mani Inc, Japan). After irrigation of the root canal with normal saline, a K-file (Dentsply-Maillefer, Ballaigues, Switzerland) with a compatible diameter was introduced into the root canal and the canal length was determined at 1 mm from the apex or root bevel (15). A K-file size #15 was introduced into the root canal and 1-2 mL India ink was injected with an insulin syringe into the orifice until the ink leaked from apical foramen. The ink was reapplied after diffusion and drying as reported by Silva *et al. *(15).

The roots were then randomly divided into 3 groups.

Group I (30 canals): the root canals were prepared manually with K-files (Dentsply-Maillefer, Ballaigues, Switzerland) up to a file size #25 and “step back” up to size #35.

Group II (30 canals): the root canals were instrumented with rotary Flex Master (VDW, Munich, Germany) instruments. At first, root canal entrances were enlarged with the Orifice Shaper “Introfile” (VDW, Munich, Germany) until the root canal middle third was reached. Crown-down preparation was performed with a 64:1 speed gear reduction handpiece of Japan (NSK Compatible) as follows: 25/04 until resist-ance was felt and 25/02 at the working length.

Rotary files were discarded after five times of use (14).

**Figure 1:**
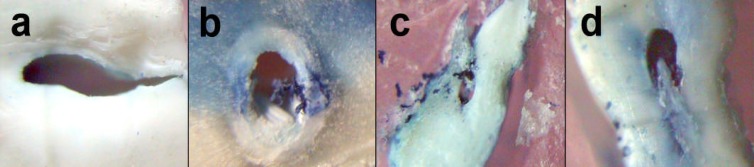
a) score 0, b) score 1, c) score 2, and d) score 3

Group III (8 canals): root canals were not instrumented and considered as control group.

In groups I and II, the root canals were prepared by the same operator. Normal saline was used for irrigation. The instrumentation time was measured for both techniques and the results were analyzed with student’s t-test. The teeth were cleared for cleaning efficacy analysis *i.e.* the teeth were placed separately in jars with a lid, containing 10% chloridric acid for 3 days (14). The acid was renewed every 24 hours until the teeth were completely decalcified. The teeth were washed under running water for 8 hours and dehydrated in 70% alcohol (for 16 hours, changed every hour), 90% alcohol (for 3 hours, changed every hour) and 96% alcohol (for 3 hours, changed every hour). After dehydration, the teeth were placed in methyl salicylate (15). At first the canals were separated from CEJ and were cut at 1 mm above the working length (2 mm upper than apex or root bevel) with a #11 scalpel, so that the apical section could be observed. Then the roots were cut from the mid part of the remaining canal (middle section). After clearing, each section was placed on a 1.5×2 inch red wax for easy observation. The removal of India ink from the cervical, middle, and apical thirds was analyzed with a stereoscopic with ×40 of magnification and scored: 0=total cleaning (Figure 1a); 1= more than 50% ink removal (Figure 1b); 2= less than 50% ink removal of total intra-canal space (Figure 1c); and 3= no ink removal (Figure 1d). An endodontist and oral pathologist, who were not informed about the groups (blind), were asked for interpretation of the sections at the same time. The results were analyzed statistically with the Mann-Whitney *U *and Friedman tests.

## RESULTS

At ×40 magnification the prepared canal walls showed variable amounts of remnant ink in canals. The score of ink distributions at coronal, middle and apical thirds are shown in Figure 1a, Figure 1b and Figure 1c according to the type of instrumentation and tooth position within jaw. No ink removed (Figure 1d) was detected in the negative control group. Mann-Whitney *U* test showed no statistically significant differences in cleaning efficacy of walls at mid and apical thirds of two groups (P=0.84 and 0.87 respectively); but the coronal third showed significantly better cleanliness in group 1 than 2 (P<0.001).

In group 2, there were no differences between the coronal, mid and apical thirds of the roots using the Friedman test (P=0.84). In group 1, the coronal thirds scored significantly better than the mid and apical thirds using the Friedman test (P=0.005 and 0.007 respectively).

**Figure 2:**
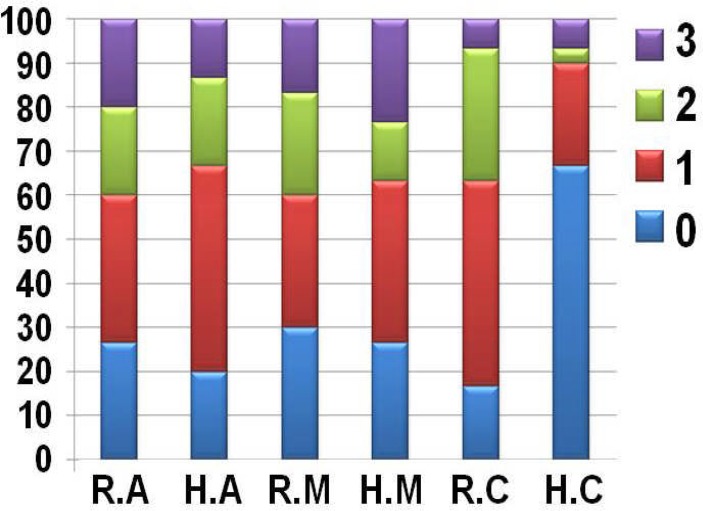
Distribution of cleaning efficacy (%) at apical (A), middle (M), and cervical (C) thirds by hand (H) and rotary (R) instrumentation

Overall the coronal, mid and apical thirds of both groups received more scores of 0 and 1 than 2 and 3. The distribution of cleaning efficacy at three thirds is shown in Figure 2.

The mean time spent for rotary root canal preparation and hand preparation were 2.07±0.49 minutes and 5.55±2.77 minutes, respectively. The difference between the two times was significant (P<0.001).

## DISCUSSION

Several factors contribute to the clinical success of pulpectomy, such as biomechanical cleaning (18), type of restoration (19), number of visits (1,18) and root canal filling material (20). Chemo-mechanical preparation of the root canal includes both mechanical instrumentation and canal irrigation, and is principally directed toward the elimination of microorganisms from the root canal system (21). Canal preparation is one of the most important phases of primary root canal treatment and is mainly aimed at the debridement of the canals (2).

The mean time spent for the instrumentation of groups 1 and 2 were 5.55 and 2.07 minutes respectively concurring with Barr *et al.*, Silva *et al.* and Mortazavi *et al*. (14,15,17). Observed mean instrumentation time of this study was lower than the one reported by Mortazavi *et al*.; their study was carried out *in vivo* study and was therefore more time consuming (17).

Versumer *et al.* proposed two separate numerical evaluation scales for debris and smear layer scoring (22). We used four scores based on another previous study (15).

Considering cleanliness there were no differences in apical and mid third of the roots between the two groups. This correlates with Silva *et al.* study that showed no significant differences in each third between the two groups; the number of rotary files used for preparation in our study however, was lower. Although Mortazavi *et al.* assessed clinical success rates, they also found no significant differences between rotary and hand instrumen-tation (17).

Some studies have reported clinical success with different types of rotary instrumentation without comparing them with hand preparation (14,16). In the coronal third of the roots, hand instrumentation showed better cleanliness than rotary instrumentation. This result did not agree Silva *et al. *(15). Although there were no statistical differences between the three thirds of the roots prepared by rotary instruments, coronal thirds of the roots prepared with hand were significantly different from the other two sections. This may be due to the operators’ tendency to place hand instruments further coronally; while the rotary preparation path is not affected by the operator.

The abrupt cervical constriction and dentinal shelf covering the canal orifice should be removed to improve the straight-line access and reduce the risk of instrument separation (16). Hence utilizing the Introfile at the coronal third was necessary to remove any impediments to gain further progress into the root canal and avoid lateral perforation or over-instrumentation of the inner root structure of the middle section. Root canals which were prepared by rotary files produced a conical pathway allowing effortless entrance of obturating paste and therefore less overfilling.

Investigations carried out permanent dentition highlighted the limited ability of endodontic instruments to clean the root canal and reinforce the importance of antibacterial irrigation for enhanced disinfection of the root canal system (21). Even Cohen *et al.* believed debridement of the primary root canal is more often accomplished by chemical means than by mechanical means (2). For meticulous evaluation of cleaning efficacy we did not use dissolving-denaturizing agents such as NaOCl. Siqueira *et al.* found that instrumentation combined with saline irrigation mechanically removed more than 90% of bacteria in the root canal (23). When simple saline was used as an irrigant, a tenfold to 1000-fold reduction of the bacterial load through mechanical instrument-ation was demonstrated (2).

Because many pulpal ramifications cannot be reached mechanically, copious irrigation during cleansing and shaping must be maintained. The authors support the view that both chemical and mechanical cleaning affects root canal cleanliness. Furthermore, a key factor in the architecture of the rotary files may be their flute design (24).

Since the "critical zone" is the apical 3 mm of the root canal system in permanent teeth (25), the real effect of less coronal cleanliness on the success rate of pulpectomy should be assessed.

## CONCLUSION

Clinically, time efficacy in primary molar endodontics, especially with the unpredictability and difficulty of canal morphology, is invaluable. The young patient and parents will appreciate every minute saved with Flex Master rotary file. With respect to modified design and easy handling, incorporating a nonhazardous irrigant such as saline or chlorhexidine is essential for a successful outcome.
